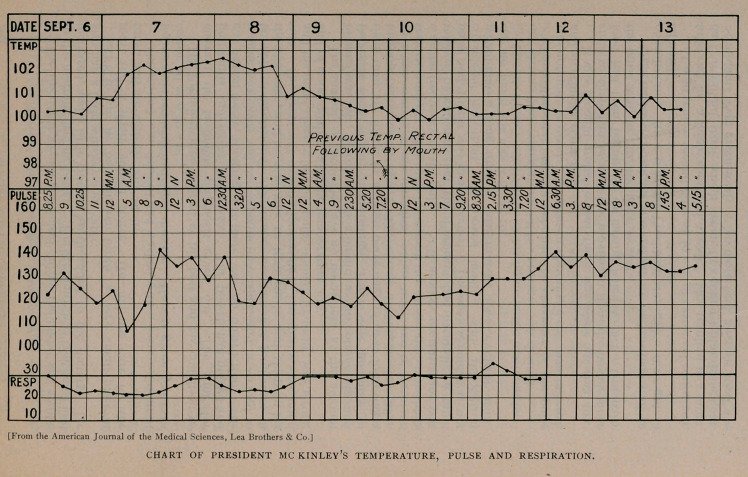# The Official Report on the Case of President McKinley

**Published:** 1901-11

**Authors:** P. M. Rixey, Matthew D. Mann, Herman Mynter, Roswell Park, Eugene Wasdin, Charles McBurney, Charles G. Stockton

**Affiliations:** Buffalo; Buffalo; Buffalo; Buffalo; Buffalo; Buffalo; Buffalo


### Special Contribution.

### The Official Report on the Case of President McKinley.

SURGICAL HISTORY.

PRESIDENT WILLIAM McKINLEY, was shot, by Leon F. Czolgosz, in the Temple of Music, at the Pan-American Exposition, Buffalo, N.Y., at about 7 minutes past 4 on the afternoon of Friday, September 6, 1901. Two shots were fired. One bullet struck near the upper part of the sternum, and the other in the left hypochondriac region. The President was immediately conveyed to the Emergency Hospital on the exposition grounds by the motor ambulance, where he arrived at 4.18. Dr. G. McK. Hall and Mr. Edward C. Mann, medical student, of the house staff, were in charge of the ambulance, medical student T. F. Ellis being the driver.

On arrival at the hospital, President McKinley was at once placed upon the table in the operating room and undressed. During the removal of his clothing a bullet fell out and was picked up by Mr. Ellis. Dr. Hall placed a temporary antiseptic dressing over the wounds, and Mr. Mann ordered a nurse to administer 0.01 gm. of morphin and 0.002 gm. of strychnin hypodermically.

Dr. Herman Mynter, who had been telephoned from police headquarters to report immediately at the exposition hospital, was the first surgeon to arrive, at 4.45 o'clock. At that time Drs. P. W. Van Peyma and Joseph Fowler, of Buffalo, and Dr. Edward Wallace Lee, of St. Louis, were present. Dr. Mynter brought with him Dr. Eugene Wasdin, of the United States Marine Hospital Service.

Dr. Mynter inspected the President's wounds, and immediately saw their serious nature. He told the President that it would be necessary to operate, and at once set about making preparations, aided by the house staff and nurses, and Dr. Nelson W. Wilson, Sanitary Officer of the Exposition, who at that time assumed charge of the hospital in the absence of Dr. Roswell Park, the Medical Director of the Exposition. The President's pulse on the arrival of Dr. Mynter was 84; he had no particular pain in the abdomen, and no apparent loss of liver dulness. He was evidently slightly under the influence of the morphin.

Dr. Matthew D. Mann arrived at the hospital at 5.10 p.m., having been telephoned for by Mr. John G. Milburn. He was followed, 5 minutes later, by Dr. John Parmenter.

An examination was at once made, followed by a short consultation between Drs. Mann, Mynter and Wasdin, which resulted in the decision to operate at once. The necessity for the operation was explained to President McKinley, and he gave his full consent. Immediate operation was decided upon because of the danger of possible continued internal hemorrhage and of the escape of gastric or intestinal contents into the peritoneal cavity, and because the President's pulse was getting weaker. Moreover, the daylight was rapidly failing. Dr. Roswell Park, who, by virtue of his office, had he been present would have performed the operation, was at Niagara Falls, and although a special train had been sent for him, it was uncertain when he would arrive.

Dr. Mann was selected to do the operation, with Dr. Mynter as his associate, by the common consent of the physicians present and at the request of Mr. Milburn, president of the Pan-American Exposition, who stated that he had been requested by President McKinley to select his medical attendants. Dr. Mann selected Drs. Lee and Parmenter as assistants.

At 5.20 Dr. Mann directed the administration of ether to President McKinley, and requested Dr. Wasdin to administer it. Ether was chosen as being, on the whole, the safer anesthetic. While the anesthetic was being given the surgeons who were to take part in the operation prepared their hands and arms by thoroughly scrubbing with soap and water, and immersing them in a solution of bichloride of mercury.

The operation began at 5.29. Dr. Mann stood upon the right-hand side of the patient, with Dr. Parmenter on his righthand side. Dr. Mynter stood upon the lefthand side of the patient, and on his right was Dr. Lee. To Drs. Parmenter and Lee were assigned the duties of sponging and the care of the instruments. Dr. P. M. Rixey, U. S. N., President McKinley's family physician, having been detailed by the President to accompany Mrs. McKinley to the Milburn home, did not arrive until 5.30, when he gave very efficient service by guiding the rays of the sun to the seat of the operation by aid of a hand-mirror, and later by arranging an electric light. Dr. Roswell Park arrived just as the operation on the stomach was completed, and gave his aid as consultant. Mr. E. C. Mann had charge of the needles, sutures and ligatures. Mr. Simpson, medical student, was at the instrument tray.

The nurses, under the charge of Miss A. C. Walters, superintendent of the hospital, were Miss M. E. Morris and Miss A. D. Barnes, with hands sterilised; Miss Rose Baron, Miss M. A. Shannon and Miss L. C. Dorchester, assistants, and Miss Katherine Simmons attending the anesthetiser.

Besides those immediately engaged in the operation, there were present Drs. P. W. Van Peyma, Joseph Fowler, D. W. Harrington and Charles G. Stockton, of Buffalo, and Dr. W. D. Storer, of Chicago.

### THE OPERATION.

President McKinley took the ether well, and was entirely under its influence in 9 minutes after the beginning of the anesthetisation. The abdomen was carefully shaved and scrubbed with green soap, and then washed with alcohol and ether and the bichlorid solution.

Inspection showed two wounds made by the bullets. The upper one was between the second and third ribs, a little to the right of the sternum. The use of a probe showed that the skin had not been penetrated, but that the bullet had probably struck a button or some object in the clothing which had deflected it. The lower wound made by the other bullet—a 32 caliber—was on a line drawn from the nipple to the umbilicus. It was about half-way between these points, and about 5 cm. to the left of the median line. A probe showed that this wound extended deeply into the abdominal walls, and that the direction was somewhat downward and outward.

An incision was made from the edge of the ribs downward, passing through the bullet wound and nearly parallel with the long axis of the body. A deep layer of fat was opened, and followed by incision of the fascia and muscles to the peritoneum. After cutting through the skin, a piece of cloth, undoubtedly a bit of the President's clothing, was removed from the track of the bullet, a short distance below the skin.

On opening the peritoneum, the finger was introduced and the anterior wall of the stomach palpated. An opening was discovered which would not quite admit the index finger. This opening was located near the greater curvature of the stomach, and about 2 cm. from the attachment of the omentum; its edges were clean-cut and did not appear to be much injured.

The stomach was drawn up into the operation wound, and the perforation very slightly enlarged. The finger was then introduced and the contents of the stomach palpated. This was done to see if the stomach contained food, and also with the hope that possibly the bullet might be in the stomach. The stomach was found to be half full of liquid food, but no evidence of the ball was discovered. In pulling up the stomach a small amount of liquid contents escaped, together with a good deal of gas. The tissues around the wound were carefully irrigated with hot salt solution and dried with gauze pads. The perforation in the anterior stomach wall was then closed with a double row of silk sutures (Czerny-Lembert). The sutures were not interrupted with each stitch, but four stitches were introduced before the ends were tied. The loop was then cut off and the suture continued. About eight stitches were used in each row. The silk used was fine black silk, the needle being a straight, round sewing needle.

In order to examine the posterior wall of the stomach, it was necessary to enlarge the incision, which now reached about 15 cm. in length. The omentum and transverse colon were pulled well out of the abdomen. The omentum was enormously thickened with fat and very rigid. In order to reach the back wall of the stomach, it was necessary to divide about 4 inches of the gastrocolic omentum, the cut ends being tied with strong black silk in two masses on each side. In this way the stomach could be drawn up in the operation wound, and the bullet wound in its posterior wall reached. This opening was somewhat larger than that in the anterior wall of the stomach, and had frayed and blood-infiltrated edges. Its exact location was impossible to determine, but it appeared to be near the larger curvature.

This opening was closed in the same way as the anterior wound, but with great difficulty, as the opening was down at the bottom of a deep pocket. A short curved surgical needle was necessary here. Little or no gastric contents appeared around this opening, but after it had been closed the parts were carefully irrigated with hot salt solution.

The operation on the stomach being now finished, Dr. Mann introduced his arm so as to palpate carefully all the deep structures behind the stomach. No trace of the bullet or of the further track of the bullet could be found. As the introduction of the hand in this way seemed to have a bad influence on the President's pulse, prolonged search for further injury done by the bullet or for the bullet itself was desisted from. The folds of the intestine which had been below the stomach were inspected for injury, but none was found. The entire gut was not removed from the abdomen for inspection, as the location of the wound seemed to exclude its injury. To have made a satisfactory search for wounds in the President's back, it would have been necessary to have entirely eviscerated him. As he was already suffering from shock, this was not considered justifiable, and might have caused his death on the operating table.

Before closing the abdominal wound, Dr. Mann asked each of the surgeons present, whether he was entirely satisfied that everything had been done which should be done and whether he had any further suggestions to make. Each replied that he was satisfied. The question of drainage was also discussed. Dr. Mynter was in favor of a Mikulicz drain being placed down behind the stomach-wall. Dr. Mann, with the concurrence of the other surgeons, decided against this as being unnecessary.

As the last step in the operation, the tissues around the bullet track in the abdominal wall were trimmed, in order to remove any tissue which might be infected. The abdominal wound was then closed with seven through-and-through silkworm-gut sutures, drawn only moderately tight, the superior layer of the fascia of the rectus muscle being joined with buried catgut. The edges of the skin were brought together by fine catgut sutures. Where the bullet had entered there was slight gaping of the tissues, but it was not thought advisable to close this tightly, as it might allow of some drainage. The wound was then washed with hydrogen dioxide and covered with aristol powder and dressed with sterilised gauze and cotton, which were held in place with adhesive straps. Over all was put an abdominal bandage.

The President bore the operation very well. The time from the beginning of the administration of the anesthetic until its discontinuance was exactly an hour and 31 minutes; the operation was completed at 6.50 p.m., having lasted from the time of the first incision an hour and 21 minutes. At the beginning of the operation President McKinley's pulse was 84. At 5.38, 0.002 gm. of strychnine was administered hypodermically. At 5.55 the respiration was 32 and the pulse 84—both good in character. At 6.09 the pulse was 88. At 6.20 it was 102, fair in character; respiration, 39. At 6.22, 1.50 gm. of brandy was administered hypodermically. At 6.48 the pulse was 124, the tension good, but quick; respiration, 36. At 7.01, after the bandage was applied, the pulse was 122 and the respiration 32. At 7.17, 0.004 gm. of morphine was administered hypodermically.

At 7.32 the patient was removed from the hospital in the ambulance. Dr. Rixey asked Drs. Park and Wasdin to go in the ambulance, as his duty called him to go at once to inform Mrs. McKinley of her husband's condition and to prepare a room for his reception. Drs. Mann and Mynter, with friends of the President, followed in carriages immediately after. President McKinley had not then recovered from the anesthetic. He bore the journey to Mr. Milburn's house exceedingly well, but it was found necessary to give him a small hypodermic injection of morphine during the transit, as he was becoming very restless. On arrival at the house of Mr. Milburn, 1168 Delaware Avenue, he was removed from the ambulance on the stretcher, and carried to a room in the northwest corner of the house, where a hospital bed had been prepared for him.

### REMARKS ON THE OPERATION.

BY MATTHEW D. MANN, M. D.

The difficulties of the operation were very great, owing partly to the want of retractors and to the failing light. The setting sun shone directly into the room, but not into the wound. The windows were low and covered with awnings. After Dr. Rixey aided us with a hand mirror, the light was better. Toward the end of the time a movable electric light with reflector was put in use. The greatest difficulty was the great size of President McKinley's abdomen and the amount of fat present. This necessitated working at the bottom of a deep hole, especially when suturing the posterior wall of the stomach.

The operation was rendered possible and greatly facilitated by a good operating table and the other appliances of a hospital, and by the presence of many trained nurses and assistants. Still, the hospital was only equipped for minor emergency work, and had but a moderate supply of instruments. Unfortunately, when called I was not told what I was wanted for, and went to the exposition grounds entirely unprepared. Dr. Mynter had his large pocket case, the contents of which were of great use.

As has already been noted, further search for the bullet was rendered inadvisable by the President's condition. The autopsy shows that it could not have been found, and that the injuries inflicted by the bullet after it passed through the stomach, were of such a nature as to render impossible and unnecessary any further surgical procedure. A bullet after it ceases to move does little harm. We were often asked why, after the operation, we did not use the *x*-ray to find the bullet. There were several reasons for this. In the first place, there were, at no time any signs that the bullet was doing harm. To have used the *x*-ray simply to have satisfied our curiosity would not have been warrantable, as it would have greatly disturbed and annoyed the patient, and would have subjected him also to a certain risk. Had there been signs of abscess-formation, then the rays could and would have been used.

My reason for not draining was that there was nothing to drain. There had been no bleeding nor oozing; there was nothing to make any discharge or secretion; the parts were presumably free from infection, and were carefully washed with salt solution. As there was no peritonitis and the abdomen was found post mortem to be sterile, we may safely conclude that no drainage could have been provided which would have accomplished anything. My experience teaches me never to drain unless there is a very decided indication for it, as a drain may do harm as well as good.

In conclusion, I wish to thank all the gentlemen who so kindly and skilfully assisted me. They were all surgeons of large experience in abdominal surgery, and their aid and advice were most valuable. Especially I wish to acknowledge my great obligation to my associate, Dr. Mynter. Not only was he an assistant, but he was much more, and helped me greatly by his skill and, as a consultant, with his good judgment and extensive knowledge of abdominal work. Although called first, he waived his claim, and generously placed the case in my hands, willingly assuming his share of the responsibility.

The anesthetic was most carefully administered by Dr. Wasdin, and the knowledge that he had charge of this very important duty relieved me of any anxiety on that score.

In the eventful week that followed the operation, Dr. Park and Dr. McBurney were towers of strength in helping to decide the many difficult questions which came up.

Dr. Rixey was in constant charge of the sick-room, aided later by Dr. Wasdin, who was detailed for this special duty. Both were unremitting in their care, and faithful to the end.

Dr. Stockton helped us in the last three days with the highest skill and best judgment.

Never, I am sure, under like circumstances, was there a more harmonious or better-agreed band of consultants. That our best endeavors failed was, I believe, no fault of ours; but it must be an ever-living and keen regret to each one of us, that we were not allowed the privilege of saving so noble a man, so attractive a patient, and so useful a life.

### THE AFTER-TREATMENT.

When put to bed the President was in fair condition: pulse, 127; temperature, 100.6°; respiration, 30. The nurses on duty were Miss K. R. Simmons and Miss A. D. Barnes, from the Emergency Hospital. Soon after his arrival, at 8.25, he was given morphine, 0.016 gm., hypodermically. There was slight nausea. The pulse soon improved. During the evening the patient slept at intervals, vomiting occasionally, but rallied satisfactorily. A slight discoloration of the dressings was noted at 10.45. There was occasional and slight pain. Ninety c.c. of urine was voided, and an enema of salt solution given and retained.

*Second Day, Saturday, September 7.*

After midnight the patient slept a good deal; he was free from pain and quite comfortable.

At 6 a.m., the temperature was 102°; pulse, 110; respiration, 24.

Gas in large quantities was expelled from the bowels. A saline enema was given as before. Miss Simmons and Miss Barnes were replaced by Miss Maud Mohan and Miss Jane Connolly. Miss E. Hunt, of San Francisco, Cal., Mrs. McKinley's nurse, also rendered assistance, and Miss Grace Mackenzie, of Baltimore, Md., arrived September 9, and was detailed for regular duty. P. A. Eliot, J. Hodgins and Ernest Vollmeyer, of the U. S. A. Hospital Corps, were detailed as orderlies.

During the forenoon, 0.01 gm. of morphine was administered hypodermically.

At 1.15 p.m., a saline enema of 500 c.c. was given. As the pulse was rising, 0.06 gm. of fluid extract of digitalis was injected hypodermically.

The President rested quietly until 6.30 p.m., when he complained of intense pain in the pit of the stomach, and was given 0.008 gm. morphine sulphate hypodermically. He was very restless, but after being sponged rested again.

At 6.30 p.m., the pulse was 130; temperature, 102.5°; respiration, 29.

During the day the digitalis, morphine and saline enemas were kept up at regular intervals; 4 gm. of somatose was added to the water at 10.30 p.m. At 11.15 p.m. the President passed from the bowels 240 c.c. of a greenish colored fluid and some particles of fecal matter.

The total amount of urine for 24 hours was 270 c.c.

#### FIRST URINALYSIS, BY DR. H. G. MATZINGER.

| | |
|---|---|
| Quantity | 30 cc. |
| Color | dark amber. |
| Reaction | strongly acid. |
| Urea | 0.028 gm. per 1 c.c. of urine. |
| Albumin | a trace. |
| Phosphates and chlorides | normal. |
| Sugar | none. |
| Indican | very small amount. |

*Microscopic Examination.*—The sediment obtained by centrifuge shows a large amount of large and small epithelial cells with some leukocytes and occasional red cells. There is a comparatively large number of hyaline casts, principally small, with some finely granular ones; also an occasional fibrinous one. The amount of sediment is large for the quantity of urine submitted. There were no crystals in the sediment.

*Third Day, Sunday, September 8.*

During the early morning the President slept a good deal, but was restless, and at times confused and a little chilly. On the whole, he passed a fairly good night.

He expelled a little gas and brown fluid from the rectum. The digitalis was continued, and at 7.45 a.m, 0.002 gm. of strychnine were given hypodermically. At 8.20 a.m. he was clear and bright, with the pulse strong and of good character.

The wound was dressed at 8.30, and found in a very satisfactory condition. There was no indication of peritonitis. Pulse, 132; temperature, 102.8°; respiration, 24.

The dressing on the wound was changed because there was some exudation. The bullet track was syringed out with hydrogen dioxide. There was very little foaming, and there were no signs of pus.

At 10.40 a.m., following an enema of epsom salts, glycerine and water, he had a small stool with gas, and another at noon. He was less restless and slept a good deal.

At noon Dr. Charles McBurney joined the medical staff in consultation, having been summoned by Dr. Rixey.

During the day he continued to improve; he slept 4 or 5 hours and his condition was satisfactory.

At 4.45 p.m., he was given a teaspoonful of water by the mouth; also an enema of sweet oil, soap and water. He passed slightly colored fluid with some little fecal matter and mucus. After this he had a small quantity of water by the mouth, and at 6.20 p.m. a nutritive enema of egg, whisky and water, which was partly retained. Digitalis and strychnine were both given during the evening.

At 9 p.m. the President was resting comfortably. The pulse was 130; temperature, 101.6°; respiration, 30.

Four hundred and twenty c.c. of urine was passed during the day.

SECOND URINALYSIS.

| | |
|---|---|
| Quantity | 450 c.c |
| Color | amber, slightly turbid |
| Reaction | strongly acid. |
| Specific gravity | 1.026. |
| Urea | 0.038 gm. per c.c. of urine. |
| Albumin | mere trace. |
| Sugar | none. |
| Indican | abundant. |
| Sulphates | increased. |
| Phosphates | somewhat increased. |
| Chlorides | somewhat increased. |

*Microscopic Examination.*—Microscopic examination of sediment obtained by centrifuge shows fewer organic elements. Some large and small epithelial cells and some leukocytes. Casts are not so abundant as yesterday and are *principally* of the small finely granular variety. There is a marked diminution in small renal epithelial cells.

Quite a quantity of large crystals of uric acid and bacteria are present.

### *Fourth Day, Monday, September 9.*

Codeia was substituted for morphia, as the pain was less. Digitalis and strychnine were stopped. Nutritive enemas were given at 3.20 a.m., at 4.30 and 10 p.m. Hot water was taken quite freely by the mouth.

Attempts to get good movement of the bowels were successful at noon, when he had a large, light-brown partly-formed stool. This followed a small dose of calomel and a high enema of oxgall.

On the whole, the President's condition improved steadily during the day. He slept a good deal and was fairly comfortable. There was no pain on pressure over the abdomen.

THIRD URINALYSIS.

| | |
|---|---|
| Quantity received | 540 c c. |
| Color | amber, slightly turbid. |
| Specific gravity | 1.026, |
| Albumin | a trace |
| Indican | not so abundant as yesterday. |
| Urea | 0.047 gm. per c.c. of urine. |
| Chlorides and phosphates | about normal. |
| Sulphates | still somewhat high. |
| Sugar | none. |

*Microscopic Examination.*—Microscopic examination of sediment obtained by centrifuge shows a decrease in the amount of organic elements and an increase of amorphous urates, but fewer crystals of uric acid. Casts are fewer and only the small granular and large hyaline varieties. The proportion of casts is greater. There are very few epithelial cells, mostly of renal type. A large number of cylindroids are found.

### *Fifth Day, Tuesday, September 10.*

Soon after midnight the President had a high enema of soap and water, which was expelled, together with some fecal matter. He took hot water frequently, and slept a good deal.

On awakening he felt very comfortable, and his mind was clear and cheerful. The nutritive enemas were kept up, and water given by the mouth. Had two small stools during the day. The only medicine given was one hypodermic of codeia phosphate, 0.015 gm.

In the evening the dressings were examined, and as there was considerable staining from the discharge, it was thought best to remove four stitches and separate the edges of the wound. A

little slough was observed near the bullet track, covering a space nearly an inch wide, the thickness of the flaps. The separation seemed to extend down to the muscle. The surfaces, except those mentioned, looked healthy, but not granulating. It was supposed that the infection of the wound occurred either from the bullet or from the piece of clothing carried into the wound at the time of the shooting. The parts were thoroughly washed with hydrogen dioxide and packed lightly with gauze, and held together with adhesive straps.

*Sixth Day, Wednesday, September 11.*

The blood count made by Dr. Wasdin in the evening was as follows:

| | |
|---|---|
| Leukocytes | 6,752 |
| Red cells | 3,920,000 |

A little after midnight, Wednesday morning, the patient was given 4 c.c. of beef juice, the first food taken by the stomach. It seemed to be very acceptable. Nutritive enema was given at 2 a.m.; later there was a yellow stool.

From 4 to 8 c.c. of beef juice was given every 1 to 2 hours during the day. The rectum was becoming irritable, and did not retain the nutritive enemas well.

At 10 a.m. the remaining stitches were removed, the wound separated and dressed. It seemed to be doing well. Most of the sloughing tissue had separated.

The patient slept much during the day, and expressed himself as feeling very comfortable. The only medicine administered was one hypodermic of strychnine.

In the evening he was changed to a fresh bed. Nutritive enemas were continued.

Urine was passed much more freely—750 c.c. in 24 hours.

FOURTH URINALYSIS.

| | |
|---|---|
| Quantity | 82 c.c. |
| Color | amber, clear. |
| Specific gravity | 1.027. |
| Reaction | strongly acid. |
| Albumin | a trace. |
| Indican | abundant. |
| Urea | 0.04 gm. per 1 c.c. of urine. |
| E. phosphates and chlorides | normal. |
| Sulphates | still a little high. |

*Microscopic Examination.*—Microscopic examination of sediment obtained by centrifuge, shows a marked diminution in amount of organic elements, but a great increase in uric acid crystals.

There are very few epithelial cells—mostly of renal type.

There are fewer casts—small and large hyaline—some finely granular.

Cylindroids are more abundant.

[From the American Journal of the Medical Sciences, Lea Brothers & Co.]

CHART OF PRESIDENT MC KINLEY'S TEMPERATURE, PULSE AND RESPIRATION.

*Seventh Day, Thursday, September 12.*

The President slept a good deal during the night, and awoke in the morning feeling better. The beef juice was continued and increased, and a little chicken broth added to the dietary. He also had a little whiskey and water.

At 8.30 a.m. he had chicken broth, a very small piece of toast and a small cup of coffee. He did not care for the toast, and ate scarcely any of it.

The wound was dressed and washed with a weak solution of iodine and then with hydrogen dioxide. He was given 30 c.c. of castor oil at 9.20 a.m.

The President now seemed at his best and his condition to warrant the favorable prognosis given out. The time for peritonitis and sepsis had passed. The bowels had moved and gas passed freely, showing that there was no obstruction. The tongue was clear, and the appetite increasing; and he seemed to be able to digest food. There was no pain nor tenderness in the abdomen, and he was able to turn easily and sleep on his side. The urine was steadily increasing. His spirits were good and his mind clear, while his pulse, though frequent, was strong and of good quality, and the temperature low.

The analysis of the urine gave no uneasiness as the amount of urea was fair; there was no albumin worth considering, and the casts were rapidly diminishing. There were no more of them than are found in a large percentage of cases following a long operation under ether. The excess of indican was taken to mean merely some intestinal indigestion, and to be of no serious import. The only symptom to cause any uneasiness was the frequency of the pulse. Still, anxiety on this score was relieved by knowing that the President had naturally a rapid pulse and that it was easily excited. The open wound was not considered important. It looked healthy, and, although it would take a long time to heal, in itself it was evidently causing no harm nor was it likely to.

Dr. McBurney left Buffalo for his home in the morning, having arranged to return at once if his presence was desired.

Toward noon it was noticed that the character of the pulse was not quite so good. Infusion of digitalis, 8 c.c., was ordered, and strychnine, 0.002 gm.

It was thought probable that there was some intestinal toxemia, as there had been no free movement from the bowels since food had been begun, the oil having failed to act. Gradually the pulse went to 130, and grew weaker.

Dr. Charles G. Stockton was added to the medical staff in consultation. At 7 p.m. the President was given 0.20 gm. of calomel.

At 9.30 p.m. a second dose of 30 c.c. of castor oil was given, followed by a high enema of oxgall. This resulted in a large, dark semifluid stool, which seemed to exhaust him somewhat. Stimulants were given freely. No more beef juice or food was given. The pulse grew rapidly worse, but at midnight there seemed some improvement, as bulletin 33 shows. At 11 p.m. 420 c.c. of normal salt solution was given simultaneously.

FIFTH URINALYSIS.

| | |
|---|---|
| Quantity | 132 c.c. |
| Color | light amber, very turbid. |
| Specific gravity | 1.025. |
| Reaction | acid. |
| Albumin | mere trace, if any. |
| Indican | less. |
| Urea | 0.044 gm. per 1 c.c. of urine. |
| Sulphates | about normal. |
| E. phosphates | much increased. |
| Chlorides | normal. |

*Microscopic Examination*—Microscopic examination of sediment obtained by centrifuge, shows fewer organic elements than the last examination. There is less uric acid and a large amount of amorphous phosphates. Renal casts, about as in the last examination, with very few cylindroids.

*Eighth Day, Friday, September 13.*

At midnight the pulse was fairly good, 132. Strychnine and whiskey were given at intervals, and hypodermics of camphorated oil.

The wound had been dressed regularly in the manner described three times a day. At 9 a.m. the dressing was changed, and a mixture of balsam of Peru and glycerine put in on gauze after the douching.

Stimulants were continued as before, but more freely. Coffee, 45 c.c., and clam broth, 60 c.c., were given; also liquid peptonoids.

At 8.30, 1.50 gm. of adrenalin was given hypodermically, and repeated at 9.40.

At 10 a.m., nearly 2 pints of normal salt solution were given under the skin, and a pint containing adrenalin at 6 p.m. Nitroglycerine and camphor were also injected at various times, together with brandy and strychnine.

Stimulants as detailed above were used freely all day.

3.30 p.m. Pulse growing weaker.

5.00 p.m. Oxygen given and continued for some hours.

6.30 p.m. Last bulletin, No. 39.

At 6.35 p.m., and again at 7,40, morphine was given hypodermically, as he was very restless and seemed to be suffering.

9 00 p.m. Heart sounds very feeble.

The President continued to sink, becoming weaker and weaker.

At 10.00 p.m., the oxygen was discontinued. The heart sounds were very feeble and consciousness lost.

The President died at 2.15 a.m., September 14.

Drs. E. J. Janeway and W. W. Johnston, who, at the request of Dr. Rixey, had been summoned in consultation, arrived too late, but were present at the autopsy. Dr. McBurney also returned on Friday afternoon.

SIXTH URINALYSIS.

| | |
|---|---|
| Color | amber, turbid, with phosphates. |
| Quantity | 252 c.c. |
| Reaction | acid. |
| Specific gravity | 1.023. |
| Albumin | mere trace, if any. |
| Urea | 0.047 gm. per 1 c.c. urine |
| Indican | a trace. |
| E. phosphates | increased. |
| Chlorides | normal. |
| Sulphates | a little high. |

*Microscopic Examination.*—Microscopic examination of sediment obtained by centrifuge, before and after clearing, shows no change from yesterday's sample. Casts, hyaline and granular, both large and small, comparatively few. Cylindroids, a few. Crystals, large amount of uric acid, some sodium urate, and in the untreated specimen a large amount of amorphous deposit, principally of phosphates There are a few epithelial cells, small, granular. Occasional red cells and leukocytes.

### REPORT ON THE AUTOPSY.[1]

BY HARVEY R. GAYLORD, M. D.,

Pathologist to the New York State Pathological Laboratory.

Ordinary signs of death: ecchymosis in dependent portions of the body. Rigor mortis well marked. Upon the surface of the chest, to the right of the midsternal line, a spot 1 cm. in diameter, dark-red in color, with a slight crust formation covering it, 5.5 cm. from the suprasternal notch; from the right nipple, 10 cm.; from the line of the right nipple, 8 25 cm. Surrounding this spot, at which point there is an evident dissolution of the continuity of the skin, is a discolored area of oval shape extending upward and to the right. In its greatest length it is 11 cm.; and in its greatest width, 6 cm. It extends upward in the direction of the right shoulder. The skin within this area is discolored, greenish-yellow and mottled.

The surface of the abdomen is covered with a surgical dressing, which extends down to the umbilicus and upward to just below the nipples. The innermost layer of cotton is covered or stained with balsam of Peru and blood. On removing this dressing, a wound parallel to, and somewhat to the left of, the median line, is exposed, inserted in which are two layers of gauze, likewise impregnated with balsam of Peru. The wound is 14.5 cm. in length, and is open down to the abdominal muscles. The layer of abdominal fat is 3.75 cm. in thickness. The appearance of the fat is good, a bright yellow in color. No evidence of necrosis or sloughing. In the left margin of the surgical wound, lying 1 cm. to the right of a line drawn from the umbilicus to the left nipple, 15.5 cm. from the nipple and 16.5 cm. from the umbilicus is a partly healed indentation of the skin, and an excavation of the fat immediately beneath it (this is the site of the entry of the

1. The autopsy was performed by Drs. Gaylord and Matzinger.

bullet), extending down to the peritoneal surface. On making the median incision, starting from the suprasternal notch and extending to a point just below the symphysis, the subcutaneous fat is exposed, which is of bright yellow color and normal appearance, except in an area which corresponds superficially to the area of discoloration described as surrounding the wound upon the chest wall. This area marks the site of a hemorrhage into the subcutaneous fat. The remainder of the subcutaneous fat is firm and measures 4.75 cm. in thickness on the abdominal wall. On opening the sheath of the right rectus muscle, it is seen to be of dark-red color. (Culture taken from ecchymotic tissue under the upper bullet hole and from between the folds of the small intestine. Three tubes from each locality on agar and gelatin )

On opening the abdominal cavity, the parietal surface of the peritoneum is exposed, and is found to be covered with a slight amount of bloody fluid; is perfectly smooth and not injected. The great omentum extends downward to a point midway between the umbilicus and the symphysis. It is thick, firm; its inferior border is discolored by coming in contact with the intestines. Below the umbilicus a few folds of intestines are exposed. These are likewise covered with discolored blood, after the removal of which the peritoneal surface is found to be shiny On the inner aspect of the abdominal wound the omentum is found to be slightly adherent to the parietal peritoneum, and can be readily separated with the hand from the edge of the wound. At this point the omentum is somewhat injected. This adhesion to the omentum is found to extend entirely around the abdominal wound. The parietal peritoneum immediately adjacent to the inner aspect of the abdominal wound is ecchymotic.

On removing the subcutaneous fat and muscles from the thoracic wall, the point which marks the dissolution of continuity of the skin upon the surface, is found to lie directly over the margin of the sternum and to the right side between the second and third ribs. There is no evidence of ecchymosis or injury to the tissues or muscles beneath the subcutaneous fat. On making an incision through the subcutaneous fat, directly through the wound upon the chest, a small cavity is exposed about the size of a pea just beneath the skin which is filled with fluid blood The subcutaneous tissue underlying the area of discoloration on the surface of the chest wall shows hemorrhagic infiltration.

On removing the sternum, the lungs are exposed, and do not extend far forward. A large amount of pericardial fat is exposed. Pleural surface on both sides is smooth. There are no adhesions on either side within the pleural cavities. The diaphragm on the right side extends upward to a point opposite the third rib in the mammary line. No perceptible amount of fluid in either pleural cavity. On opening the pericardial cavity, the surface of the pericardium is found to be smooth and pale. The pericardium contains approximately 6 c.c. of straw-colored, slightly turbid fluid. (Some taken for examination.)

On exposing the heart, it is found covered with a well-developed panniculus. The heart measures, from the base to the apex, on the superficial aspect, 10.5 cm. The right ventricle is apparently empty The heart feels soft and flaccid. On opening the left ventricle, a small amount of dark-red blood is found The muscle of the left ventricular wall is 1.5 cm. in thickness; dark reddish-brown in color; presents a shiny surface. The average thickness of the pericardial fat is 3.5 mm. (Cultures made from the auricle.) The left auricle contains but a small amount of dark currant-colored blood. The mitral valve admits three fingers. The right ventricle, when incised in the anterior line, is found to be extremely soft; the muscular structure is 2 mm. in thickness. The panniculus measures 7 mm. The muscle is dark-red in color; very shiny, and the pericardial fat invades the muscular wall at many points.

On opening the right auricle, it is found to be filled and distended by a large currant-colored clot, which extends into the vessels. The tricuspid orifice admits readily three fingers. The coronary arteries are patulous and soft; no evidence of thickening.

Lungs are gray color and contain a moderate amount of coal-dust pigment. Slight amount of frothy fluid escapes from the bronchi; but the pulmonary tissue is crepitant and free from exudate.

On unfolding the folds of intestine, there is no evidence of adhesion until a point just beneath the mesocolon is reached, when, on removing a fold of small intestine, a few spoonfuls of greenish-gray thick fluid flows into the peritoneal cavity.

On the anterior gastric wall is an area to which a fold of the gastrocolic omentum is lightly adherent. On breaking the adhesion, there is found a wound about midway between the gastric orifices, 3.5 cm. in length, parallel with the greater curvature of the stomach, 1.5 cm. from the line of omental attachment. This wound is held intact by silk sutures. There is no evidence of adhesion at any other point on the anterior wall. The gastric wall surrounding the wound just mentioned for a distance of 2 cm. to 3 cm. is discolored, dark greenish-gray in appearance, and easily torn. On exposing the posterior wall of the stomach from above, along its greater curvature, the omentum is found to be slightly. adherent, a line of silk ligatures along the greater curvature of the stomach marking the site where the omentum had been removed. On throwing the omentum downward, the posterior gastric wall is exposed. On the posterior wall, a distance of 2 cm. from the line of omental attachment, is a wound approximately 2 cm long, held intact by silk sutures. The gastric wall surrounding this wound is discolored. On the surface of the mesocolon, which is posterior to the gastric wall at this point, is a corresponding area of discoloration, the portion coming directly in contact with the wound in the gastric wall being of dull gray color. The remainder of the surface of the posterior wall of the stomach is smooth and shiny. Beyond the surgical wound in the posterior wall of the stomach is found an opening in the retroperitoneal fat, large enough to admit two fingers. This opening communicates with a track which extends downward and backward as far as the finger can reach. The tissues surrounding this track are necrotic. On removing the descending portion of the colon, a large irregular cavity is exposed, the walls of which are covered with gray, slimy material, and in which are found fragments of necrotic tissue. Just at the superior margin of the kidney is located a definite opening which forms the bottom of the track traced from the stomach. On stripping the left kidney from its capsule, it is found that the superior portion of the capsule is continuous with the cavity. The weight of the left kidney is 5 oz. 1 gm. The kidney is readily stripped from its capsule; is dark red; the stellate veins are prominent, and along its greater curvature are numerous dark red depressions. On the superior aspect of the kidney is a protrusion of the cortex, dark red in color, and in this protrusion is a laceration 2 cm. long, extending across the superior border, approximately at right angles to the periphery of the kidney and from before backward. On incising the kidney, the cortex and medulla are not easily distinguishable from one another; both are of rose red color, the cortex measuring approximately 6 mm. in thickness. The vessels in the pyramids of Ferrein are very prominent. Beneath the protruding portion of the surface, the cortex is dark red in color. This discoloration extends downward in pyramidal form into the medulla. The laceration of the surface marks the apex of the protrusion of the kidney substance. Between the spleen and the superior aspect of the kidney is a necrotic tract which extends down and backward, and ends in a blind pocket. The tract which included the superior aspect of the kidney can be traced into the perinephritic fat to a point just above the surface of the muscles of the back.

The necrotic cavity which connects the wound on the posterior wall of the stomach and the opening adjacent to the kidney capsule is walled off by the mesocolon, and is found to involve an area of the pancreas, approximately 45 mm. in diameter and extending about half-through the organ. This organ at its center forms part of the necrotic cavity. Through its body are found numerous minute hemorrhages and areas of gray softening, the size of a pea or smaller. These are less frequent in the head portion of the pancreas.

A careful examination of the track leading down toward the dorsal muscles fails to reveal the presence of any foreign body. After passing into the fat, the direct character of the track ceases; and its direction can be traced no farther. The adjoining fat and the muscles of the back were carefully palpated and incised, without disclosing a wound or the presence of a foreign body. The diaphragm was carefully dissected away, and the posterior portion of the thoracic wall likewise carefully examined. All fat and organs which were removed, including the intestine, were likewise examined and palpated, without result.

The great amount of fat in the abdominal cavity and surrounding the kidney rendered the search extremely difficult.

The right kidney is imbedded in a dense mass of fat; capsule strips freely; it weighs 5 ounces; measures 11.5 cm.; substance is soft; cortex is 6 mm. in thickness; rose-red in color; cut surface slightly dulled. There are a few depressions of the surface, and the stellate veins are prominent.

The liver is dark-red in color; the gall-bladder distended. The organ was not removed.

The autopsy continued for a longer period than was anticipated by those who had charge of the President's body, and we were requested to desist seeking for the bullet and terminate the autopsy. As we were satisfied that nothing could be gained by locating the bullet, which had apparently set up no reaction, search for it was discontinued.

*Anatomic Diagnosis.*—Gunshot wound of both walls of the stomach and the superior aspect of the left kidney; extensive necrosis of the substance of the pancreas; necrosis of the gastric wall in the neighborhood of both wounds; fatty degeneration, infiltration and brown atrophy of the heart muscle; slight cloudy swelling of the epithelium of the kidneys.

A matter of no inconsiderable embarrassment to us arose in the objection of our removing sufficient portions of the tissues for examination. We were able to secure only two small fragments of the stomach wall; tissue from around the wound upon the chest wall; a portion of fat from the wall of the necrotic cavity; a small piece of each kidney, that of the left kidney including the portion involved by the original wound; and pieces of heart-muscle from the right and left ventricles. The microscopic examination of these tissues follows:

The piece of retroperitoneal fat, where it forms part of the necrotic cavity, is seen on section to be covered with a thick gray deposit, which has an average thickness of from 4 mm. to 6 mm. Beneath this and separating it from the fat, is a well-defined area of hemorrhage from 1 mm. to 2 mm. in thickness. The appearance of this piece of tissue is characteristic of the fat tissue surrounding the entire cavity. A section made perpendicular to the surface and stained with hematoxylin-eosin, shows the following characteristics: Under low power there is no evidence of round-celled infiltration between the fat cells, or of fat necroses. The surface of the tissue which, in the microscopic specimen was covered by a layer of grayish material, proves, under low power, to consist of a partly organised fibrinous deposit. At the base of this deposit is evidence of an extensive hemorrhage, marked by deposits of pigment. The surface of the membrane is of rough and irregular appearance, and contains a large number of round cells with deeply stained nuclei. Under high power the organisation of the membrane may be traced from the base toward the surface. The portion immediately adjacent to the fat tissue consists of a network of fibrin enclosing large numbers of partly preserved red blood corpuscles. In many areas the red blood corpuscles are broken down and extensive deposits of pigment are found. Extending into the fibrin structure of the membrane are numerous typical fibroblasts and round cells. In some regions pigment is evidently deposited in the bodies of large branching and spindle cells. Here and there, included in the membrane, are the remains of fat cells, and toward the surface of the membrane a large number of round cells scattered through the interstices of the membrane. There are but few polymorphonuclear leukocytes. Here and there in the membrane are fragments of isolated fibrous connective tissue with irregular contours and an appearance suggesting that they are fragments of tissue which have been displaced by violence and included in the fibrin deposit. The fibrin in the superficial layers of the membrane is formed in hyaline clumps. The organisation along the base of the deposit is comparatively uniform.

Sections stained with methylen blue, carbol-thionin and Gram's method were carefully examined for the presence of bacteria, with negative results. Even upon the surface of the membrane there are no evidences of bacteria.

The section of the left kidney, including the triangular area of hemorrhage described in the macroscopic specimen, reveals the following appearances: (Section hardened in formalin, stained with hematoxylin-eosin.) Examined macroscopically, section represents a portion of a kidney cortex made perpendicular to the surface of the cortex, and including an area of hemorrhage into the substance of the cortex 1 cm. in length, measured from the capsular surface downward, and presenting a width of from 5 mm. to 6 mm. The capsular surface has apparently been torn.

Under low power the margins of the preparation are found to consist of well preserved kidney structure. There is a slight amount of thickening of the interstitial tissue, and occasional groups of tubules are affected by beginning cloudy swelling. The glomeruli are large and present a perfectly normal appearance. As we approach toward the center of the preparation, occasional glomeruli are met with in which the capillary loops are engorged and the adjacent tubules contain red blood-corpuscles. A short distance farther, the kidney structure becomes entirely necrotic. Here and there the remains of tubules may be made out, and these are infiltrated with cells. The necrotic area presents a rough, net-like structure. As we approach toward the surface of the kidney, we find that the necrosis becomes more marked. There is the merest suggestion of kidney structure, its place being taken by disintegrated red blood-cells and leukocytes, embedded in a well-defined fibrinous network. There is great distortion of the kidney structure about the periphery of the necrotic area. In this region a considerable amount of pigment is also found in the necrotic tissues.

Under high power, the characteristics of the necrotic tissues may be better observed. The kidney structure is broken up and torn into irregular fragments, infiltrated by red blood corpuscles and leukocytes. In the portion of the necrotic mass beneath the capsule, the kidney structure is practically obliterated and is replaced by a network of fibrin, which includes large numbers of red blood cells and leukocytes. Scattered through the entire necrotic area are frequent deposits of pigment. In the deeper portions of the necrotic area, the margins of the fibrin deposit are invaded by fibroblasts from the connective tissue structure of the kidney. The organisation in these areas is, however, slight.

Sections stained with methylen blue and Gram's method and carefully examined under oil immersion, fail to reveal the presence of any organisms. In preparations stained with methylen blue, the deposits of pigment may be readily observed. Section of the same tissue hardened in Hermann's solution and examined for fat, shows the presence of numerous fat droplets within the epithelium of the tubules which are adjacent to the area of necrosis. In the portions of the preparation more widely distant from the area of necrosis no fat is present.

Section of the right kidney hardened in formalin and stained with hematoxylin-eosin reveals the presence of areas in which slight parenchymatous degeneration of the epithelium in the uriniferous tubules may be noted. These areas are not extensive and are confined to single groups of tubules. The interstitial connective tissue of the organ seems to be slightly increased in amount, but there is no well-defined round-celled infiltration. An occassional hyaline glomerulus is to be met with in these cases surrounded by increased connective tissue The epithelium of the kidney tubules, aside from those in which the parenchymatous degeneration is present, is well preserved. The nuclei are well stained; protoplasm, finely granular.

A fragment of the stomach wall taken from the immediate neighborhood of the anterior wound is in a condition of complete necrosis. The nuclei of the cells are scarcely demonstrable. The epithelial surface is recognised with difficulty. At its base are apparently a few round cells. Examination of the blood vessels reveals nothing characteristic. There is apparently no evidence of thrombosis A section made through the gastric wall at some distance from the wound reveals the well-preserved muscular structure of the gastric wall, which presents no characteristic alterations. Superficial portions of the epithelium have apparently been affected by post-mortem digestion. However, in one portion of the preparation, the epithelium is intact, and shows distinct evidence of marked round-celled infiltration between the glandular structures. The blood vessels contained blood-corpuscles with the usual number of leukocytes.

The fragments of heart-muscle which were removed from the right and left ventricular walls, were examined in the fresh state, and exhibited a well-defined fatty degeneration of the muscle fibers, and in the case of the right ventricular wall, an extensive infiltration between the muscle fibers, of fat, was apparent. Sections from these fragments of muscle hardened in Hermann's solution, are taken for examination. A fragment of muscle from the right ventricular wall was removed at a point where the fat penetrated deeply into the muscular structure, the ventricular wall at this point showing an average thickness of 2.5 mm. Under low power, the muscle fibers are separated into bundles by masses and rows of deeply stained fat cells. The muscle fibers are seen to contain groups of dark brown granules lying in the long axes of the cells. Under high power, these are resolved into extensive groups of dark brown pigment arranged around the nuclei. The muscle fibers are slender, the cross and longitudinal striation is well defined. Examined near the margin of the preparation, where the osmic-acid fixation has been successful, all of the muscle fibers are found to contain minute black spherical bodies, extending diffusely through all the muscle fibers about the entire margin of the preparation. These fine fat droplets are present in sufficient amount to speak of an extensive diffuse fatty degeneration of the muscle fibers. Where the large fat cells have separated the muscle fibers, these are found to be more atrophic than those in the central portions of the larger bundles.

The examination of the section through the healed bullet wound on the chest walls reveals nothing of importance. The dissolution of continuity is filled in by granulation-tissue, and there is evidence of beginning restoration of the epithelium from the margins. Stains for bacteria give negative results.

In summing up the macroscopic and microscopic findings of the autopsy, the following may be stated: The original injuries to the stomach wall had been repaired by suture, and this repair seems to have been effective. The stitches were in place, and the openings in the stomach-wall effectually closed. Firm adhesions were formed both upon the anterior and posterior walls of the stomach, which reinforced these sutures. The necroses surrounding the wounds in the stomach do not seem to be the result of any well-defined cause. It is highly probable that they were practically terminal in their nature, and that the condition developed as a result of lowered vitality. In this connection there is no evidence to indicate that the removal of the omentum from the greater curvature and the close proximity of both of these wounds to this point, had any effect in bringing about the necrosis of the gastric wall, although circulatory disturbances may have been a factor. The fact that the necrotic tissue had not been affected by digestion strongly indicates that the necrosis was developed but shortly before death. The excavation in the fat behind the stomach must be largely attributed to the action of the missile. This may have been the result of unusual rotation of a nearly spent ball, or the result of simple concussion from the ball passing into a mass of soft tissues. Such effects are not unknown. The fact that the ball grazed the superior aspect of the left kidney, shown by the microscopic investigation of that organ, indicates the direction of the missile, which passed in a line from the inferior border of the stomach to the tract in the fat immediately posterior to the kidney. There was evidence that the left adrenal gland was injured.

The injury to the pancreas must be attributed to indirect, rather than direct, action of the missile. The fact that the wall of the cavity is lined by fibrin, well advanced in organisation, indicates that the injury to the tissues was produced at the time of the shooting. The absence of bacteria from the tissues, indicates that the wound was not infected at the time of the shooting, and that the closure of the posterior gastric wound was effectual. The necrosis of the pancreas seems to us of great importance. The fact that there were no fat necroses in the neighborhood of this organ, indicates that there was no leakage of pancreatic fluid into the surrounding tissues. It is possible that there was a leakage of pancreatic fluid into the cavity behind the stomach, as the contents of this cavity consisted of a thick, grayish fluid, containing fragments of connective tissue. In this case the wall of fibrin would have been sufficient to prevent the pancreatic fluid from coming in contact with the adjacent fat. The extensive necrosis of the pancreas would seem to be an important factor in the cause of death, although it has never been definitely shown how much destruction of this organ is necessary to produce death. There are experiments upon animals upon record, in which the animals seem to have died as a result of not very extensive lesions of this organ. One experiment of this nature reported by Flexner (*Journal of Experimental Medicine*, Vol. II.) is of interest. The fact that concussion and slight injuries of the pancreas may be a factor in the development of necrosis is indicated by the researches of Chiari (*Zeitschrift fur Heilkunde*, Vol. XVII., 1896, and *Prager Med. Wochenschr.*, 1900, No. 14), who has observed (although a comparatively rare condition) extensive areas of softening and necrosis of the pancreas, especially of the posterior central portion which lies directly over the bodies of the vertebra, where the organ is most exposed to pressure or the effects of concussion. The wound in the kidney is of slight importance, except as indicating the direction taken by the missile. The changes in the heart, as shown by the macroscopic inspection and the microscopic examination, indicate that the condition of this organ was an important factor. The extensive brown atrophy and diffuse fatty degeneration of the muscle, but especially the extent to which the pericardial fat had invaded the atrophic muscle fibers of the right ventricular wall, sufficiently explain the rapid pulse and lack of response of this organ to stimulation during life.

### REPORT ON THE BACTERIOLOGIC EXAMINATION.

BY HERMAN G. MATZINGER, M. D.,

Bacteriologist to the New York State Pathological Laboratory.

It is obvious that the short space of time which has elapsed since the death of the President has hardly been sufficent to prepare a complete and thorough bacteriologic report. This report contains all the observations which have been made up to this time:

On September 11, during the life of the President, cultures were made by Dr. Wasdin from the base of the abdominal wound and from dressings removed at the same time. These were submitted to me for examination, and showed the presence of the ordinary pus organisms: Staphylococcus pyogenes aureus and S. cereus albus, with a gas-forming bacillus which, in pure anerobic culture on glucose gelatin, forms small, pearly, translucent colonies, with no liquefaction. In litmus milk it produces acid, but no coagulation. Morphologically, it is apparently a capsulated, short bacillus, which takes stains poorly, and which does not stain by Gram's method. Inoculated into the ear vein of a rabbit, which was killed immediately afterward, it produced, after twenty-four hours in the body of the rabbit, a marked accumulation of gas in the organs, and again grew out in pure culture. As yet the organism is not fully identified.

None of these cultures showed streptococci. A bacterium which appears to be one of the proteus group was, however, isolated which does not stain by Gram, and appears in varying forms, sometimes small oval, and again quite rod-shaped and in short chains. Sometimes it is surrounded with a slimy covering, which remains clear like a capsule when the organism is stained. On slanting agar, it produces a whitish, slimy growth, which gradually runs to the bottom of the slant and produces an odor of decomposition. On gelatin, it grows very slowly with slight and slow indication of liquefaction. In litmus milk, it produces acid and rapid coagulation.

At the time of the autopsy, September 14, inoculations were made by myself. From the base of the wound, there was again obtained a number of pus organisms, principally a white staphylococcus and the bacterium described above, but no streptococci. Cultures made from the peritoneal surface of the intestines were entirely negative. Cultures made from the under surface of the omentum near the colon, were entirely negative, both with and without oxygen. Cultures from the blood of the right auricle were likewise negative. A very careful and extensive search for microörganism in the contents of the necrotic cavity, behind the stomach, reveals nothing but a short, stumpy bacterium, which, as far as the work has been carried at present appears to belong to the proteus group, and is very like proteus hominis capsulatus, described by Bordoni and Uffreduzzi.

Morphologically, it is not uniform, and sometimes appears almost encapsulated, being surrounded by material that does not stain; is quite refractory to Gram, and produces an odor of decomposition as it grows. It does not liquefy gelatin rapidly and grows slowly, as a glistening white elevated surface growth which slowly sinks; but on agar in the thermostat it grows very rapidly, as a moist, grayish-white, translucent mass. Colonies on gelatin plates have a clean circumference, are granular and quite refractive. In litmus milk it produces acid and rapid coagulation. Animal experiments are still incomplete and cannot be published at this time.

It must be stated that there is occasion for suspecting that this may be a contamination, either from the outer wound or elsewhere, because, quite unavoidably, the technic in obtaining the material and cultures from the necrotic cavity was not absolutely correct.

Cultures made from the small area of broken-down tissue under the chest wound at the time of the autopsy, grew what appears to be staphylococcus epidermidis albus, described by Dr. Welch.

The slimy, gray, necrotic material from the cavity above the transverse mesocolon behind the stomach, was carefully examined microscopically, with the result that very few microörganisms were found in the fresh state, and no recognisable tissue elements of any kind, no leukocytes or pus-corpuscles, but an abundance of crystals which appeared more like fatty acid than fat crystals. It contained no free hydrochloric acid, and was alkaline in reaction. Experiments as to its digestive power were negative. About 2 c.c. of this material was injected into the space behind the stomach of a dog (still living), with no results except quite an elevated temperature for three or four days. Other animal experiments are still incomplete.

It might be well to state here that the bacteriologic examination of the chambers and barrel of the weapon used, as well as the empty shells and cartridges, ordered by the District Attorney, was entirely negative, except that from a loaded cartridge there was grown an ordinary staphylococcus and a mold. The chemical examination of the balance of the loaded cartridges, made by Dr. Hill, chemist, was also negative.

The absence of known pathogenic bacteria, particularly in the necrotic cavity, warrants the conclusion that bacterial infection was not a factor in the production of the conditions found at the autopsy.

---

The foregoing report has received the approval of, and is issued by, the undersigned, the medical staff attending the late President, William McKinley.

P. M. Rixey,
Matthew D. Mann,
Herman Mynter,
Roswell Park,
Eugene Wasdin,
Charles McBurney,
Charles G. Stockton.

Buffalo, October 12, 1901.

Note by the Editor—The report of the medical staff in attendance upon President McKinley is printed in full, with the exception of the bulletins. These were all printed in the October issue of the Journal and may be referred to with interest in relation to this report.

## Figures and Tables

**Figure f1:**